# Association between environmental factors and BMI: evidence from recent immigrants from developing countries

**DOI:** 10.1186/s41043-019-0174-4

**Published:** 2019-07-05

**Authors:** Bhagyashree Katare, Sanchita Chakrovorty

**Affiliations:** 1 Department of Agricultural Economics, Purdue University, 640 Krannert Building, 403 W State Street, West Lafayette, IN 47906 USA; 2 Department of Agricultural Economics, Purdue University, 631 Krannert Building, 403 W State Street, West Lafayette, IN 47906 USA

**Keywords:** Immigrants, BMI, Obesity

## Abstract

**Background:**

To study the association between the surrounding local environmental factors and the body mass index of immigrants in the USA.

**Methods:**

We use the New Immigrant Survey, to study the association of obesity prevalence in a region on body mass index. We consider local obesity rate as an outcome of the local environmental factors. Using ordinary least squares, three versions of equations are estimated to quantify the contribution of individual-level, acculturation, and environmental effects on immigrants’ body mass index.

**Results:**

We find statistically significant results for the correlation of local obesity rate and body mass index. For every 1% increase in the obesity rate, the body mass index levels increase by 0.182 kg/m^2^. Evidence also suggests dietary assimilation in immigrants is influenced by local environmental factors and that dietary change affects body mass index of female immigrants.

**Conclusions:**

Immigrants’ body mass index increase with the increase in the local obesity rate of the region where they reside.

**Electronic supplementary material:**

The online version of this article (10.1186/s41043-019-0174-4) contains supplementary material, which is available to authorized users.

## Background

Rising rate of obesity is a policy concern, as it is associated with increased health risks and health costs. Obesity has been linked to chronic diseases such as coronary heart diseases, type 2 diabetes, cancer, hypertension, and depression [1, 2]. These diseases account for 21% of health care cost in the USA [3]. Obesity is largely viewed as an individual outcome originating from individual choices [4–6]. Changes in individual behavior, such as increases in fast-food consumption [7], increases in the consumption of sweetened beverages [8], unhealthy eating habits [9], physical inactivity [10], and increases in television viewing time [11], have been associated with the rise in obesity. Research has also found that in addition to unobserved social networks, there are unobserved confounding contextual factors that are responsible for the spread of obesity [12], but isolating the effect of environmental factors has been challenging. Our research attempts to isolate the contextual environmental factors and studies their association with the obesity prevalence in the immigrant population.

Individual’s local environment is a function of factors such as the socio-economic status in the area, access to supermarkets and grocery stores, concentration of fast-food restaurants, physical infrastructure, economic policy, cultural environment, and proximity to recreational centers and parks. These characteristics define choices available to individuals and in turn affect their health. As these environmental factors affect the health outcomes of the local population, they also in turn influence the local obesity rate (LOR) of that region [13–19]. For this paper, we consider LOR as the long-run outcome of the process by which inhabitants affect their environment; specifically, the social, cultural, and physical environments of a region, and in turn, are affected by it.

The LOR of a region is defined as the percent of obese adults in a given region. Even though the obesity rate in the USA is 35.7%, there is heterogeneity in the regional obesity rate [20]. For instance, Colorado, the least obese state in the country, has an obesity rate of 22.6%, whereas West Virginia, one of the most obese states in the country, has an obesity rate of 38.1% [21].

According to the healthy immigrant theory, immigrants on arrival are healthier than the native population and with time their health converges to that of the natives [22–24]. Immigrants are a unique population in that before arrival, they are not exposed to the local environment of the region, to which they are immigrating. Upon arrival in the USA, they are potentially influenced by the habits and lifestyle of their new region of residence. Indeed, two identical immigrants may face very different local environments if they move to different parts of the USA and thus may adopt different lifestyles and dietary habits because they are exposed to different environments. These choices influence the health of the immigrants [25, 26]. We seek to understand if LOR, a function of the varying environmental factors, have a varying influence on the body mass index (BMI) levels of the immigrants. Consider two new immigrants who have similar characteristics. One of them immigrates to Mississippi where the obesity rate is 35.5%, while the other individual immigrates to Utah, where the obesity rate is 25.7% [21]. Environmental factors that affect the obesity rate in Mississippi are different from those in Utah. We ask the question, how does exposure to different environments affect the health status of otherwise similar individuals?

Immigrants undergo a process of acculturation when they move into a new environment of the host country. Adoption to the new social culture and dietary environment has an effect on immigrant’s health [22]. This process of adoption to a new lifestyle may result in acquiring new health behavior and dietary habits thus resulting in unhealthy outcomes [26–31]. Research shows that with the passage of time, immigrants gain more weight [25, 32–34] and who arrive at a younger age are at higher risk of obesity than those who arrive at an older age [35].

The paper adds to the literature by studying the association of local environmental factors and immigrants’ BMI levels. Consider this: if people were to choose their place of residence independent of the LOR and the factors that influence it, then the regression of people’s BMI on the LOR would yield causal results. However, this is a very strong assumption, mainly for two reasons. First, people’s choice of a place of residence is a choice of that place’s local environmental characteristics, which also influence the LOR. Second, the place of residence has a contextual effect on people. They adapt to the local environment prevalent in the surrounding area, which is influenced by the observable and unobservable preferences of other people living in that area. As immigrants are born abroad, they are not exposed to the cultures of the regions of the USA. Hence, they might not be aware of the local characteristics of the different regions. If we assume that new immigrants choose to live in a region independent of the LOR and the factors that drive it, then we can causally identify the role that environmental factors play in obesity prevalence.

According to McDonald and Kennedy [25], association of immigrant with their ethnic social network in the host country accelerates their assimilation process with host country’s culture and lifestyle, in turn influencing immigrants’ health. Katare and Beatty [36] find that international students who chose to attend a university in a higher obesity region gain significantly more weight as compared to those who chose a university in a lower obesity region. They show that international students’ choice of university is independent of the local environmental factors, thus establishing an almost causal relationship between the LOR and weight gain in students. Following this literature, a modest version of our maintained assumption would be that the immigrants’ choice of location within the USA is entirely based on lowering their adjustment costs such as job opportunities and available support from relatives and earlier immigrants, and the local environmental characteristics such as prevalence of obesity, availability of recreational activities, and density of grocery stores or fast food restaurants are not considered in the decision-making process.

Current obesity epidemic in the USA is a result of changes in local environmental conditions [37] such as local food environment [19] and types of food stores [17], which influence the food choices and in turn affect the health outcomes. The proximity to obesogenic environmental factors such as availability of fast-food restaurants closer to schools or residential areas is shown to be a risk factor for obesity prevalence [38, 39]. Obesity can be considered as a part of social phenomenon connected to individuals’ social life [40]. Through the process of acculturation, immigrants interact with new culture and society in the host country. They learn new ways of life, customs, and languages. Acculturation measured by immigrants’ language skills [29], college degree [28], and a measure of English language preference [27] have been found to be a risk factor affecting the obesity trajectory.

To the best of our knowledge, this is one of the first papers that attempt to isolate the effect of local environmental characteristics on the BMI levels of immigrants. This paper extends prior work by considering how the weight gain is correlated with the LOR. We study the mechanism of obesity prevalence by considering the correlation between the immigrants’ BMI level and environmental, acculturation factors.

## Methods

We use the New Immigrant Survey, 2003 (NIS 2003), a nationally representative survey of legal immigrants to the USA, conducted in 2003–2004. It is a random sample of full cohort of immigrants who have newly acquired their legal permanent residence. The final sample construction for this paper is reported in Table 3 in Additional file 1 consisting 1189 male and 913 female immigrants. This research is reviewed by the authors’ university’s Institutional Review Board and has determined that it meets the criteria for exemption under 45 CFR 46.101(b).

BMI is used as the main outcome variable, calculated from the self-reported height and weight data available in NIS 2003. BMI range is from 12 to 65.5 kg/m^2^. LOR is our main variable of interest. State-level obesity rates for 2003 are obtained from The State of Obesity [41]. NIS 2003 data were collected in 2003–2004, and the state-level obesity rates used are for the year 2003. NIS divides the country in eight census regions and seven states. For each region in the eight census regions, we calculated the average of the local obesity rate for all the states included in that given census region. For example, the LOR mapped to the New England regions is the average of the obesity rates for Vermont, Rhode Island, New Hampshire, Massachusetts, Maine, and Connecticut. For each of the seven states, we assigned their corresponding state-level obesity rate. The details for the obesity rate calculation and assignments for each state and region are in Table 4 in the Additional file 1.

Previous research indicates that the process of acculturation varies by gender [42, 43]; hence, we perform the analysis separately for men and women. Prior work has also suggested that spread of obesity is related to the environment in which individuals live and shared environmental factors can cause the appearance of social network effects [12]. Community-level effects arise due to shared experiences. In other words, individuals living in the same area are exposed to the same fast-food restaurants, the same exercise and wellness facilities, and the same obesity rate. These factors affect the health or BMI levels of all individuals who might or might not be in one another’s social circles. When a foreign individual is introduced into a population, he/she voluntarily or involuntarily adapts to the given social and physical infrastructure.

We want to quantify the contribution of each level of variation (individual-level effects, acculturation effects, and environmental effects) on immigrants’ BMI levels. We estimate three versions of equations using ordinary least squares. Standard errors are corrected for heteroscedasticity and are clustered at the state level to correct for arbitrary within-state correlation. By controlling individual specific demographic variables, in the first specification, we estimate the influence of LOR on BMI measures of immigrants.

1 $$ {\mathbf{BMI}}_{\mathbf{i}\mathbf{s}}={\boldsymbol{\upbeta}}_{\mathbf{1}}{\left(\mathbf{local}\ \mathbf{obesityrate}\right)}_{\mathbf{i}\mathbf{s}}+{\boldsymbol{\upbeta}}_{\mathbf{2}}{\mathbf{Z}}_{\mathbf{i}\mathbf{s}}+{\mathbf{u}}_{\mathbf{i}} $$

where the LOR corresponds to the percent of adults who are obese in the state s of residence of immigrant i and *Z* is a vector of individual-level control variables corresponding to immigrant i.

In the second specification on Eq. 2, we augment the first version with acculturation variables (Acculturation_is_).

2 $$ \mathbf{BM}{\mathbf{I}}_{\mathbf{i}\mathbf{s}}={\boldsymbol{\upbeta}}_{\mathbf{1}}{\left(\mathbf{local}\ \mathbf{obesity}\ \mathbf{rate}\right)}_{\mathbf{i}\mathbf{s}}+{\boldsymbol{\upbeta}}_{\mathbf{2}}{\mathbf{Z}}_{\mathbf{i}\mathbf{s}}+{\boldsymbol{\upbeta}}_{\mathbf{3}}{\mathbf{Acculturation}}_{\mathbf{i}\mathbf{s}}+{\mathbf{u}}_{\mathbf{i}} $$

In the third specification in Eq. 3, we control for environmental variables that are likely to be correlated with the LOR:

3 $$ \mathbf{BM}{\mathbf{I}}_{\mathbf{i}\mathbf{s}}={\boldsymbol{\upbeta}}_{\mathbf{1}}{\left(\mathbf{local}\ \mathbf{obesity}\ \mathbf{rate}\right)}_{\mathbf{i}\mathbf{s}}+{\boldsymbol{\upbeta}}_{\mathbf{2}}{\mathbf{Z}}_{\mathbf{i}\mathbf{s}}+{\boldsymbol{\upbeta}}_{\mathbf{3}}{\mathbf{Acculturation}}_{\mathbf{i}\mathbf{s}}+{\beta}_{\mathbf{4}}{\mathbf{W}}_{\mathbf{i}\mathbf{s}}+\kern0.5em {\mathbf{u}}_{\mathbf{i}} $$

where *W*_is_ is a vector of environmental variables in the region of residence of immigrant i in state s.

## Results

Table 1 presents the descriptive statistics for the demographic, health, and acculturation variables of the entire sample of 1189 male (57%) and 913 female (43%) respondents separately. More male immigrants have a college degree and are employed as compared to female immigrants. The average duration of residence in the USA for the entire sample is 8 years, and 68% of immigrants are married. The highest numbers of immigrants are from Latin America followed by Asian countries. More than 55% of immigrants consider themselves to speak English “well” or “very well.” The last column in Table 1 reports the differences between male and female characteristics, and almost all the variables are statistically different, except for age and time of residence as expected.

**Table 1:** Descriptive statistics of the adult immigrants in NIS 2003

Variables	Entire sample, *N* = 2102	Male, *N* = 1189	Female, *N* = 913	Difference between male and female
Demographic variables				
Age (years)	36.11 (10.31)	35.95 (10.35)	36.33 (10.26)	− 0.83
No. of children	1.51 (1.67)	1.37 (1.61)	1.69 (1.72)	− 4.31***
Income (= income/$10,000)	3.49 (4.54)	4.04 (5.29)	2.77 (3.18)	6.45***
Duration of residence in the USA (year)	8.21 (6.90)	8.16 (6.79)	8.27 (7.06)	− 0.36
Female	–	56.57%	43.43%	–
College degree (= 1)	34.1%	38.77%	28.1%	5.11***
Married (= 1)	67.98%	71.57%	63.3%	4.03***
Employed (= 1)	80.20%	88.22%	69.76%	10.81***
Citizenship sponsored through employer (= 1)	24.73%	29.18%	18.94%	5.42***
From Asia (= 1)	32.58%	34.39%	30.23%	2.02**
From Africa (= 1)	13%	16.73%	8.21%	5.79***
From Latin America and the Caribbean (= 1)	54.37%	48.87%	61.56%	− 5.83***
Health variables				
BMI (kg/m^2^) (12 < BMI < 65.5)	25.53 (5.03)	25.77 (4.39)	25.21 (5.76)	2.57***
Health behaviors				
Currently smoking cigarettes (= 1)	23.26%	30.86%	13.36%	9.61***
Currently consuming alcohol (= 1)	41.67%	51.47%	28.91%	10.67***
Change in diet (=1)	52.52%	53.40%	51.36%	0.93
Acculturation variables				
Speaks English well/very well (= 1)	55.18%	61.98%	46.3%	7.24***
Speaks English with spouse (= 1)	26.26%	27.83%	24.21%	1.87*
Speaks English at work (= 1)	71.12%	78.38%	61.66%	5.52***
Speaks English with friends (= 1)	51.56%	57.69%	43.59%	6.47***
Education in the USA (years)	1.27 (2.77)	1.34 (2.8)	1.16 (2.73)	1.48

Standard deviation values are in parentheses when reporting averages. For continuous variables, we report mean and corresponding standard deviations in parentheses. For binary variables, we report percentages. *P < 0.1, **P < 0.05, ***P < 0.01. Statistics for BMI categories are reported in Table 6 in Additional file 1

Results from Table 2 show the estimates for the effect of LOR on the BMI of immigrants. The LOR has a significant effect on the BMI levels of all the immigrants. For every 1% increase in the LOR, the BMI of female immigrants increases by 0.182 kg/m^2^. For interpretation, consider if a person instead of immigrating to Colorado (obesity rate 22.3%), immigrated to Georgia (obesity rate 31.4%), her BMI would be 1.66 kg/m^2^ (0.182 × 9.1 = 1.66) more than her BMI in Colorado. Translating this into weight will mean that instead of immigrating to Colorado, if a person immigrated to Georgia, she would have gained almost 11.15 lbs. (5.1 kg) more weight in Georgia than she would have gained in Colorado (the reference height for these calculations is 1.75 mts and weight 50 kg). However, female immigrants drive these results. The local environmental factors and the acculturation factors have a higher and significant effect on the female immigrants as compared to the male immigrants.

**Table 2:** OLS estimates for effect of environment on BMI of immigrants (NIS 2003)

Dependent var.: BMI	Male			Female			All		
	(1)	(2)	(3)	(1)	(2)	(3)	(1)	(2)	(3)
Local obesity rate	0.107 (0.061)	0.111* (0.059)	0.089 (0.112)	0.182** (0.076)	0.175** (0.077)	0.261** (0.090)	0.148** (0.063)	0.144** (0.062)	0.162* (0.092)
Age (years)	0.028* (0.014)	0.024 (0.014)	0.023 (0.014)	0.076*** (0.022)	0.076*** (0.021)	0.073*** (0.021)	0.046** (0.017)	0.040** (0.017)	0.039** (0.017)
College degree (= 1)	− 0.186 (0.239)	− 0.174 (0.258)	− 0.143 (0.256)	− 0.828** (0.353)	− 0.891** (0.346)	− 0.883** (0.343)	− 0.552** (0.245)	− 0.517** (0.238)	− 0.478* (0.226)
Married (= 1)	0.229 (0.238)	0.389 (0.232)	0.407 (0.232)	0.814 (0.521)	0.865 (0.559)	0.850 (0.561)	0.414 (0.268)	0.505 (0.312)	0.519 (0.317)
Employed (= 1)	0.638** (0.267)	0.608** (0.262)	0.614** (0.26)	− 0.198 (0.382)	− 0.053 (0.376)	− 0.011 (0.383)	− 0.090 (0.288)	− 0.0003 (0.284)	0.018 (0.285)
Number of children	0.275** (0.118)	0.257** (0.118)	0.256** (0.11)	0.578*** (0.126)	0.546*** (0.137)	0.536*** (0.14)	0.456*** (0.061)	0.437*** (0.063)	0.431*** (0.064)
Duration of residence in the USA (years)	0.066** (0.027)	0.076** (0.026)	0.072** (0.027)	0.033 (0.025)	0.044* (0.022)	0.035 (0.023)	0.050** (0.026)	0.064** (0.024)	0.057** (0.026)
Citizenship sponsored through employer (= 1)	0.267* (0.192)	0.197 (0.198)	0.240 (0.230)	0.253 (0.431)	0.091 (0.430)	0.213 (0.443)	0.307 (0.230)	0.224 (0.234)	0.298 (0.264)
From Asia (= 1)	− 1.159*** (0.265)	− 1.206*** (0.278)	− 1.283*** (0.253)	− 2.191*** (0.667)	− 2.060*** (0.615)	− 2.412*** (0.703)	− 1.574*** (0.250)	− 1.576*** (0.250)	− 1.738*** (0.294)
From Africa (= 1)	Base	Base	Base	Base	Base	Base	Base	Base	Base
From Latin America and the Caribbean (= 1)	1.430** (0.577)	1.461** (0.555)	1.367** (0.507)	1.621** (0.743)	1.679** (0.642)	1.317** (0.601)	1.569** (0.562)	1.538** (0.525)	1.353** (0.469)
Income (= income/10000)	0.049* (0.024)	0.055** (0.022)	0.055** (0.022)	− 0.027 (0.050)	− 0.026 (0.047)	− 0.014 (0.045)	0.047*** (0.014)	0.050*** (0.014)	0.052*** (0.013)
Gender	–	–	–	–	–	–	− 0.967*** (0.197)	− 0.974*** (0.178)	− 0.976*** (0.185)
Smoker (= 1)	− 0.11 (0.257)	− 0.078 (0.260)	− 0.077 (0.261)	− 0.653 (0.731)	− 0.630 (0.744)	− 0.616 (0.763)	− 0.321 (0.192)	− 0.292 (0.185)	− 0.283 (0.188)
Alcohol consumer (= 1)	0.403 (0.245)	0.410 (0.234)	0.409* (0.227)	− 0.003 (0.270)	0.018 (0.267)	0.017* (0.279)	0.215 (0.194)	0.244 (0.192)	0.227 (0.187)
Diet change (= 1)	–	− 0.149 (0.283)	− 0.145 (0.280)	–	0.249* (0.127)	0.265* (0.141)	–	− 0.013 (0.170)	− 0.011 (0.164)
Education in the USA (years)	–	− 0.065** (0.030)	− 0.066** (0.029)	–	− 0.072 (0.043)	− 0.073* (0.041)	–	− 0.071** (0.025)	− 0.071** (0.024)
Speaks English well/very well (= 1)	–	− 0.037 (0.314)	0.059 (0.317)	–	0.736* (0.390)	0.645 (0.384)	–	0.264 (0.258)	0.255 (0.258)
Speaks English with spouse (= 1)	–	− 0.577** (0.207)	− 0.583** (0.207)	–	− 0.187 (0.259)	− 0.169 (0.256)	–	− 0.390* (0.188)	− 0.383* (0.187)
Speaks English with friends (= 1)	–	0.440 (0.275)	0.463 (0.290)	–	0.209 (0.379)	0.319 (0.369)	–	0.341 (0.251)	0.396 (0.267)
Speaks English at work (= 1)	–	− 0.190 (0.427)	− 0.215 (0.442)	–	− 0.968** (0.374)	− 0.947** (0.368)	–	− 0.689* (0.376)	− 0.698* (0.377)
Percentage of immigrant population	–	–	0.012 (0.027)	–	–	0.099*** (0.025)	–	–	0.049** (0.021)
Percentage of White population in the state	–	–	− 0.008 (0.025)	–	–	0.047* (0.022)	–	–	0.013 (0.018)
State poverty rate	–	–	− 0.002 (0.047)	–	–	− 0.007 (0.052)	–	–	− 0.002 (0.042)
Constant	20.03*** (1.187)	20.15*** (1.138)	21.16*** (4.051)	16.74*** (1.586)	16.88*** (1.329)	10.42** (3.609)	20.23*** (1.312)	20.69*** (1.138)	18.70*** (3.084)
*N*	1189	1189	1189	913	913	913	2102	2102	2102
*R* square	0.151	0.156	0.157	0.253	0.258	0.263	0.191	0.195	0.198
Individual control variables	Yes	Yes	Yes	Yes	Yes	Yes	Yes	Yes	Yes
Acculturation variables	No	Yes	Yes	No	Yes	Yes	No	Yes	Yes
Environmental variables	No	No	Yes	No	No	Yes	No	No	Yes

Standard errors are corrected for heteroscedasticity and clustered at state level. Robust standard errors are in parentheses. *P < 0.1, **P < 0.05, ***P < 0.01. Individual controls are immigrant demographic variables such as age, employment status, number of children, marital status, year, if citizenship was sponsored, and the region of origin. Acculturation variables are English-speaking proficiency, use of English while conversing at work, use of English with friends, use of English with a spouse, years of education in the USA. Environmental variables are poverty rate, percent of immigrants, and percent of White population in the state

The inclusion of acculturation variables in column 2 does not change the magnitude of the estimate (0.175 kg/m2), indicating that the acculturation variables are not correlated to the LOR. The inclusion of environmental variables increases the magnitude of the estimated coefficient for the LOR significantly by almost 43% implying that 43%, i.e., (0.261–0.182)/0.182, of the correlation between the LOR and female immigrants’ BMI level can be attributed to the heterogeneity among states rather than the heterogeneity among respondents. Result also shows that immigrants living in the USA for a longer period had higher BMI. These results are similar to the previous literature on immigrants’ BMI and duration of residence [28]. Female immigrants with well or very well English proficiency level have higher BMI. Male immigrants conversing in English with their spouses have lower BMI than those who do not converse in English.

We also estimated a model with an interaction term between LOR and duration of residence. The duration of residence was divided into two categories, short stay (time of residence ≤ 8 years) and longer stay (time of residence > 8 years). Results presented in Table 7 in Additional file 1 show that the LOR has the same effect on the BMI of female immigrants regardless of their duration of residence. The coefficients for both the interaction term are statistically similar (*P* > 0.1). This result echoes the main results that the local obesity rate influenced female immigrants’ BMI.

## Discussion and conclusions

This paper uses NIS 2003 data to examine the relationship between immigrants’ BMI and LORs. Results show that BMI among immigrants increased with an increase in the LOR. For immigrants located in environments with a higher percentage of the obese population, the local environmental characteristics have a positive association with their BMI.

Results also show that among immigrants who have been living in the USA for a longer period, there was a significant effect of environmental factors on their BMI. With the passage of time, the effect of the environment is replaced by the effect of the behavioral change. Even if people in different regions have spent the same amount of time, the influence of different environments has different effects on their BMI. This result is important because it provides additional support for our initial hypothesis that environmental factors affect BMI levels. These support the idea of policy interventions to modify the environment to provide healthier choices for the individuals. Healthier environment may motivate people in making healthier choices and may lead to positive health outcomes. Female immigrants mainly drive the results. These results are supported by the existing literature, which states that female immigrants converge to the American BMIs at a faster rate than the male immigrants [22] and that the female immigrants’ body composition is affected more as compared to men after moving to the USA. [44]. Results underline the higher health risk in female immigrants and the need to design obesity prevention policies taking into account gender and ethnic heterogeneity.

We find that acculturation measured as female immigrant’s English language skills was significantly correlated with their BMI. These results contribute to the literature that the language used at home is an important level of acculturation [45] and is a significant predictor of obesity in female immigrants [46, 47]. Similarly, college-educated female immigrants had lower BMI than those with lower levels of education, which is similar to the results from previous literature [28]. There is a possibility of interrelation between the environmental variables and the degree of acculturation in female immigrants. Factors such as employment, education, and proficiency in English language determine the dimension of acculturation through access to American lifestyle and adoption of habits such as decreased physical activity and increased consumption of high-fat energy dense food [48].

Our results contribute to the knowledge about the effectiveness of policy interventions geared toward modifying the local environment, such as state taxes on sugar-sweetened beverages, immigrant population, public transportation, density of food deserts, parks and recreation infrastructure, and outdoor recreation opportunities. However, our results do not provide information about specific environmental factors (such as concentration of fast food restaurants versus convenience stores) those may influence immigrant BMI. One main reason is our data does not allow us to define a geographical unit finer than at state level restricting to account for these environmental factors. Our results show that immigrant BMI is correlated with the percentage of immigrant population in their region and the state poverty rate. These results are supported by the previous literature on environmental factors. McDonald and Kennedy find that immigrants in areas with higher ethnic population have lower weight gain as compared to others [25]. Similarly, Kling et al. find that moving from a higher to a lower poverty neighborhood reduces the probability of being obese [49]. Katare and Beatty find that limited access to healthy food has a significant effect on weight gain in international students [36]. These studies provide evidence that environmental factors influence individual health outcomes. Our results cautiously suggest that to improve individual health outcomes, there is a need to implement policies to modify the environment. An unhealthy environment may limit the effect of individual-level interventions thus restricting the effect of these interventions on individual health behavior and choices.

We are assuming that prior to arriving in the USA, immigrants are unaware of the local conditions of their places of immigration. This assumption can fail for several reasons. For instance, it is possible that the immigrants are aware of the local environmental characteristics in a region, and this knowledge motivates them to move to a certain region. It is also possible that immigrants move to a place with a high percentage of their co-ethnic population. This may shield immigrants from the social and cultural environments in the region.

Immigrants are a unique population as they are placed in a new and unknown environment and make choices available in their foreign environment. Hence, their results might not be generalizable to other populations. Another important concern with respect to the magnitude of the coefficients is that environmental variables can vary within the states, with respect to their per-capita income, infrastructure, and population density. These varying environments within a state can create a classical error-in-variables problem and would bias the co-efficient towards zero. Change in physical activity after coming to the USA can play an important role in the weight gain mechanism for the immigrant population. Future work can be focused on exploring this relationship. Regardless of the above problems, this paper contributes both to the obesity and immigration literature by examining the influence of the local environment on the BMI levels among immigrants. Results establish a robust and non-trivial association between the local environmental factors in the form of the LOR and the BMI levels of immigrants.

## Additional files

Additional file 1: **Table S1.** Sample construction -NIS 2003 **Table S2.** Local obesity rate calculation for 15 regions **Table S3.** Descriptive Statistics for Environmental Variables **Table S4.** Descriptive Statistics for different BMI categories **Table S5.** OLS Estimates for Effect of Environment on BMI of Immigrants with interaction of local obesity rate and duration of stay (NIS 2003). (DOCX 42 kb)

## Data Availability

This study used publicly available data for New Immigrant Survey (NIS) 2003 (https://nis.princeton.edu/index.html).
