# Supplementary material for: Association between environmental factors and BMI: evidence from recent immigrants from developing countries
Source: J Health Popul Nutr. 2019 Jul 5;38:15. doi: 10.1186/s41043-019-0174-4 (PMC6612128; doi:10.1186/s41043-019-0174-4)
Supplement: Supplementary file 1 — Table S1. Sample construction -NIS 2003 Table S2. Local obesity rate calculation for 15 regions Table S3. Descriptive Statistics for Environmental Variables Table S4. Descriptive Statistics for different BMI categories Table S5. OLS Estimates for Effect of Environment on BMI of Immigrants with interaction of local obesity rate and duration of stay (NIS 2003). (DOCX 42 kb) [file 41043_2019_174_MOESM1_ESM.docx]

# Appendix

Appendix Table A.1. Sample construction -NIS 2003

| **Reason** | **Number of Affected Respondents** | **Remaining Respondents** |
| --- | --- | --- |
| Initial Sample |  | 8,573 |
| Immigrated from country other than the country of their birth | 1,496 | 7,077 |
| Missing reason of temporary living overseas during the survey | 138 | 6,939 |
| Data missing for age height, weight and units for height and weight | 402 | 6,537 |
| Missing country of origin; state of current residence; date of immigration and other demographic variables | 196 | 6,341 |
| Not from a developing country | 1,101 | 5,240 |
| Missing income information | 3,134 | 2,106 |
| Implausible height, weight response | 4 | 2,102 |

Appendix Table A.2. Local obesity rate calculation for 15 regions

| States | Local Obesity Rate (Raw) | Local Obesity Rate (Final) |
| --- | --- | --- |
| California | 23.2 | 23.2 |
| Florida | 19.9 | 19.9 |
| Illinois | 23.7 | 23.7 |
| New York | 20.9 | 20.9 |
| New Jersey | 20.1 | 20.1 |
| Texas | 24.6 | 24.6 |
| New England | | |
| Vermont | 19.6 | Average = 19 |
| Rhode Island | 18.4 |  |
| New Hampshire | 20.2 |  |
| Massachusetts | 16.8 |  |
| Maine | 19.9 |  |
| Connecticut | 19.1 |  |
| Middle Atlantic | | |
| Delaware | 24 | Average = 23.2333 |
| Maryland | 21.9 |  |
| Pennsylvania | 23.8 |  |
| District of Columbia | 20.3 |  |
| Virginia | 21.7 |  |
| West Virginia | 27.7 |  |
| South Atlantic | | |
| North Carolina | 24 | Average = 24.5667 |
| South Carolina | 24.5 |  |
| Georgia | 25.2 |  |
| East South Central | | |
| Alabama | 28.4 | Average = 26.775 |
| Mississippi | 28.1 |  |
| Tennessee | 25 |  |
| Kentucky | 25.6 |  |
| East North Central | | |
| Indiana | 26 | Average = 24.25 |
| Michigan | 25.2 |  |
| Wisconsin | 20.9 |  |
| Ohio | 24.9 |  |
| West North Central | | |
| Iowa | 23.9 | Average = 23.37143 |
| Kansas | 22.6 |  |
| Minnesota | 23 |  |
| Missouri | 23.6 |  |
| Nebraska | 23.9 |  |
| North Dakota | 23.7 |  |
| South Dakota | 22.9 |  |
| West South Central | | |
| Arkansas | 25.2 | Average = 24.8 |
| Louisiana | 24.8 |  |
| Oklahoma | 24.4 |  |
| Mountain | | |
| Arizona | 20.1 | Average = 19.875 |
| New Mexico | 20.2 |  |
| Colorado | 16 |  |
| Nevada | 21.2 |  |
| Montana | 18.8 |  |
| Wyoming | 20.1 |  |
| Idaho | 21.8 |  |
| Utah | 20.8 |  |
| Pacific | | |
| Oregon | 21.5 | Average = 21.6 |
| *Washington* | *21.7* |  |

***Source****: Stateofobesity.org (2003)*

Appendix Table A.3. Descriptive Statistics for Environmental Variables

| **Environmental Variables** | **Mean** | **Standard Deviation** |
| --- | --- | --- |
| Local Obesity Rate | 22.25 | 1.89 |
| Percentage of White Population in the State | 71.34 | 9.88 |
| Percentage of Immigrants in the State | 15.97 | 8.05 |
| Poverty Rate of the State | 11.78 | 2.23 |
| N | 2,102 | |

***Sources:***

***Percentage of White Population****: U.S. Census Bureau, Census 2000 Summary File 1, Matrices P3, P4, PCT4, PCT5, PCT8, and PCT11.*

***Percentage of Immigrants****: Value generated by Jeanne Batalova of the MPI Data Hub (Migration Policy Institute). Estimates for 2000 are from the US Census Bureau, Summary File 3, 2000 US Decennial Censuses.*

***Poverty Rate:*** *US Census Bureau, Poverty in the United States: 2001, Report P60-219, Table 4, p. 10, September 2002.*

Appendix Table A. 4. Descriptive Statistics for different BMI categories

| **BMI Categories** | **Male** | | **Female** | | **All** |
| --- | --- | --- | --- | --- | --- |
| Underweight( < 18.5 kg/m^2^) | 17.72 (0.56) | | 17.38 (0.86) | | 17.47 (0.81) |
| Normal weight (18.5 -24.9 kg/m^2^) | 22.65 (1.63) | | 21.94 (1.69) | | 22.32 (1.69) |
| Overweight (25.0 – 29.9 kg/m^2^) | 27.18 (1.42) | | 27.28 (1.39) | | 27.21 (1.41) |
| Obese (30.0 – 34.9 kg/m^2^) | 34.05 (5.79) | | 35.08 (6.51) | | 34.58 (6.18) |
| N | 1,189 | 903 | | 2,102 | |

Appendix Table A.5. OLS Estimates for Effect of Environment on BMI of Immigrants with interaction of local obesity rate and duration of stay (NIS 2003)

|  | **Male** | | | **Female** | | | **All** | | |
| --- | --- | --- | --- | --- | --- | --- | --- | --- | --- |
| **Dependent Var.:BMI** | **(1)** | **(2)** | **(3)** | **(1)** | **(2)** | **(3)** | **(1)** | **(2)** | **(3)** |
| Local Obesity Rate*duration (<8 years) | 0.149**  (0.072) | 0.154*  (0.076) | 0.145  (0.107) | 0.151*  (0.078) | 0.141*  (0.082) | 0.261**  (0.109) | 0.144**  (0.070) | 0.139*  (0.070) | 0.018*  (0.091) |
| Local Obesity Rate*duration (>8 years) | 0.049  (0.113) | 0.052  (0.113) | 0.010  (0.162) | 0.236**  (0.107) | 0.239**  (0.108) | 0.274**  (0.112) | 0.155  (0.044) | 0.155**  (0.068) | 0.143  (0.101) |
| Duration (less than 8 years) | -3.215  (2.952) | -3.417  (3.186) | -4.089  (3.050) | 1.337  (2.807) | 1.534  (2.884) | -0.241  (3.04) | -0.541  (1.707) | -0.610  (1.717) | Base |
| Duration (greater than 8 years) | Base | Base | Base | Base | Base | Base | Base | Base | 1.715  (1.467) |
| Age (Years) | 0.031**  (0.014) | 0.027*  (0.015) | 0.026  (0.015) | 0.779***  (0.022) | 0.079***  (0.021) | 0.075***  (0.021) | 0.048  (0.017) | 0.044**  (0.017) | 0.042**  (0.017) |
| College Degree (=1) | -0.186  (0.245) | -0.172  (0.270) | -0.131  (0.271) | -0.819**  (0.342) | -0.899**  (0.341) | -0.896**  (0.342) | -0.546  (0.243) | -0.524**  (0.238) | -0.486**  (0.231) |
| Married (=1) | 0.251  (0.227) | 0.398*  (0.220) | 0.451*  (0.219) | 0.826  (0.537) | 0.868  (0.151) | 0.854  (0.564) | 0.439  (0.270) | 0.516  (0.315) | 0.528  (0.318) |
| Employed (=1) | 0.628**  (0.268) | 0.597**  (0.257) | 0.607**  (0.255) | -0.187  (0.385) | -0.039  (0.378) | -0.001  (0.385) | -0.087  (0.299) | 0.005  (0.290) | 0.023  (0.290) |
| Number of Children | 0.270  (0.116) | 0.251**  (0.114) | 0.250**  (0.114) | 0.565***  (0.131) | 0.532***  (0.141) | 0.526***  (0.138) | 0.446  (0.064) | 0.427***  (0.069) | 0.421***  (0.060) |
| Citizenship sponsored through employer (=1) | 0.313  (0.190) | 0.247  (0.196) | 0.295  (0.226) | 0.283  (0.419) | 0.133  (0.421) | 0.232  (0.433) | 0.335  (0.217) | 0.257  (0.222) | 0.325  (0.250) |
| From Asia (=1) | -2.570***  (0.379) | -2.649***  (0.324) | -2.624***  (0.322) | -3.808***  (0.864) | -3.766***  (0.821) | -3.756**  (0.835) | -3.119  (0.583) | -3.114***  (0.530) | -3.090***  (0.544) |
| From Africa (=1) | -1.393**  (0.491) | -1.432*  (0.460) | 1.319***  (0.416) | -1.592**  (0.710) | -1.685**  (0.609) | -1.329**  (0.591) | -1.510  (0.501) | -1.508***  (0.461) | -1.325***  0.420 |
| From Latin America and the Caribbean (=1) | Base | Base | Base | Base | Base | Base | Base | Base | Base |
| Income (=income/10000) | 0.048**  0.021) | 0.055**  (0.019) | 0.053**  (0.416) | -0.025  (0.057) | -0.023  (0.052) | -0.013  (0.049) | 0.047 (0.014) | 0.051***  (0.013) | 0.052***  (0.013) |
| Gender | - | - | - | - | - | - | -0.945  (0.190) | -0.951***  (0.168) | -0.956***  (0.174) |
| Smoker (=1) | -0.115  (0.248) | -0.085  (0.251) | -0.087  (0.254) | -0.619  (0.732) | -0.595  (0.742) | -0.581  (0.760) | -0.297  (0.197) | -0.266  (0.188) | -0.260  (0.19) |
| Alcohol Consumer (=1) | 0.464*  (0.238) | 0.478**  (0.230) | 0.473**  (0.223) | -0.003  (0.274) | 0.014  (0.269) | 0.009  (0.277) | 0.235  (0.195) | 0.265  (0.193) | 0.246  (0.189) |
| Diet Change (=1) | - | -0.138  (0.287) | -0.136  (0.282) | - | 0.234*  (0.134) | 0.251  (0.148) | - | -0.021  (0.167) | -0.019  (0.162) |
| Education in U.S. (Years) | - | -0.074**  (0.029) | -0.075**  (0.029) | - | -0.067  (0.048) | -0.071  (0.045) | - | -0.073**  (0.025) | -0.074**  (0.025) |
| Speaks English Well/Very Well (=1) | - | 0.044  (0.336) | 0.060  (0.337) | - | 0.779*  (0.385) | 0.681*  (0.381) | - | 0.295  (0.270) | 0.277  (0.271) |
| Speaks English with spouse (=1) | - | -0.549**  (0.229) | -0.557**  (0.229) | - | -0.144  (0.267) | -0.134  (0.263) | - | -0.344  (0.201) | -0.341  (0.200) |
| Speaks English with Friends (=1) | - | 0.440  (0.301) | 0.461  (0.315) | - | 0.203  (0.378) | 0.316  (0.373) | - | 0.343  (0.258) | 0.396  (0.273) |
| Speaks English at Work (=1) | - | -0.209  (0.474) | -0.236  (0.485) | - | -0.974**  (0.373) | -0.943**  (0.370) | - | -0.689  (0.401) | -0.693  (0.401) |
| Percentage of Immigrant Population | - | - | 0.017  (0.025) | - | - | 0.100***  (0.029) | - | - | 0.051**  (0.021) |
| Percentage of White Population in the State | - | - | -0.006  (0.025) | - | - | 0.049**  (0.023) | - | - | 0.015  (0.018) |
| State Poverty Rate | - | - | 0.008  (0.049) | - |  | -0.005  (0.050) | - | - | 0.008  (0.041) |
| Constant | 23.71***  (2.363) | 24.06***  (2.555) | 24.99***  (5.185) | 17.683***  (2.441) | 17.726***  (2.276) | 11.739**  (3.924) | 22.34***  (1.244) | 22.84***  (1.258) | 19.24***  (3.244) |
| N | 1,189 | 1,189 | 1,189 | 913 | 913 | 913 | 2,102 | 2,102 | 2,102 |
| R Square | 0.153 | 0.158 | 0.160 | 0.253 | 0.258 | 0.263 | 0.191 | 0.196 | 0.199 |
| Individual Control Variables | Yes | Yes | Yes | Yes | Yes | Yes | Yes | Yes | Yes |
| Acculturation Variables | No | Yes | Yes | No | Yes | Yes | No | Yes | Yes |
| Environmental Variables | No | No | Yes | No | No | Yes | No | No | Yes |

*Note: Standard Errors are corrected for heteroscedasticity and clustered at state level. Robust Standard Errors in parentheses. *P<0.1, **P<0.05, ***P<0*
